# The Report on Poor-Law Orders: Points of Institutional Interest

**Published:** 1913-08-30

**Authors:** 


### THE REPORT ON POOR-LAW ORDERS.

### Points of Institutional Interest.

In the last issue of The Hospital we drew attention to some of the more important provisions in the Report of the Departmental Committee on Poor-Law Orders affecting the medical officers of mixed workhouses. We return to the subject to-day to comment upon some other matters of general and medical interest contained in the Report. The Draft Order has made a concession to the public sentiment voiced in the Report of the Royal Commission on the Poor Laws, 1905, by omitting the use of the words "workhouse" and "pauper." For the former the word institution is substituted, and for the latter the word inmate. The first important alteration of the old Poor-Law Orders is contained in Article 4 of the Draft Order, which prohibits the retention of children above five years of age in the mixed workhouse for a longer period than six weeks, unless the child is an inmate of the sick wards, or there are other medical reasons for its retention. This prohibition will meet with general approval. The necessity for it is shown by the fact that on January 1, 1913, excluding the sick and children under three, there still remained in workhouses 3.3 per cent. of the total number of children chargeable to the Poor Law. The Committee report that it was originally their intention to recommend that this prohibition should apply to all children above three years of age. It is a pity that this intention was modified, and that the retention of children is permitted for so long a period as six weeks. Many Boards of Guardians have made arrangements by which children over three are not received into the workhouse, but are sent direct to a children's home. This is the ideal to be aimed at, and by combined action in the case of the smaller Unions could be attained by all. In Article 8 it is provided that in any case of urgency in which the medical officer is unable to examine an inmate immediately on his admission, the master may direct that the inmate shall at once be placed in a ward appropriate for his care and treatment.

### A Dangerous Power.

This is a dangerous power to give to the master. The receiving ward should be so arranged that such cases could be properly looked after until the medical officer is able to attend. If this is not possible, the master should act only in accordance with regulations to be framed by the medical officer, and in cases of doubt after consultation with the superintendent nurse, whose special training and experience would obviously be of some assistance in the solution of difficulties.

The classification of inmates is left much more to the discretion of the Guardians than is the case under the old Orders, but the right of inmates to see their children, who may be in the workhouse, at least once a day, is restricted in the Draft Order to the mother, and in her case is to apply only to an infant under three years of age. It is difficult to see why the father should be deprived of this right.

A new regulation provides that "every case in which a lunatic, or alleged lunatic, is placed in a padded-room, or otherwise compulsorily secluded, shall be recorded in a book to be produced to every Commissioner in Lunacy inspecting the institution." This is an extension of the Local Government Board Order of September 1, 1908, which refers only to the seclusion of persons not certified to be of unsound mind. A seclusion book such as that contemplated in the new Order is already kept in many workhouses, and it is certainly desirable that its keeping should be compulsory.

### Guardians and Visiting.

The clause giving the power to a member of the Board of Guardians to visit and examine any part of the institution is reproduced from the old Orders. The only alterations made are the substitution of "at reasonable hours" for "at any time," and the introduction of a limiting clause that such visiting is to be subject to any general regulations made by the Guardians. In their Report the Committee state that it was suggested to them that the clause should be amended so as to permit a Board of Guardians to withdraw this privilege from a particular member of the Board, but that it appeared to them that the power that would thus be given to the Guardians might be so used as to defeat the objects of the clause. The risk of an elected Board acting in this way is probably slight, whereas the desirability of its possessing the power suggested is held by many experienced Poor-Law Guardians and officers. Similarly, it is to be regretted that the Committee did not decide to prohibit a male Guardian from inspecting the female wards except in the company of a female Guardian or female officer.

A new regulation requires the Guardians to cause a record to be kept showing for every inmate who is not in the sick wards, or lunatic wards, or an infant under eighteen months, every occasion on which he applies for, or is ordered, medical treatment, the nature of his complaint, and the nature of any treatment ordered. The clause does not say who is to keep this book, but it seems probable that it will fall to the lot of the medical officer.

### Changes from the Old Orders.

With regard to the duties prescribed for the medical officer, in addition to the points commented upon last week, the following changes from the old Orders may be noted: (1) He is to examine, within twenty-four hours before removal, every child or infant whom it is proposed to transfer from the institution, and certify the result of his examination; (2) he is no longer required to notify to the Local Government Board outbreaks of infectious disease in the institution, or the death from accident of an inmate who was not in receipt of relief at the time of the accident; and (3) in making all his records he is to use the terms employed in the Nomenclature of Diseases drawn up by the Joint Committee appointed by the Royal College of Physicians. The phrase in the old Order that "according to his agreement he is to supply the requisite medicine to all poor persons in the workhouse" does not appear in the proposed Order, but unfortunately the Guardians are not prohibited from entering into such agreements.

The duty of making a half-yearly report to the Guardians upon the condition of the institution, the nursing of the inmates, and any defects in either is continued. In the Order of April 4, 1868, this report had to be sent to the Poor-Law Board. Unfortunately this was rescinded by the Order of August 24, 1869. It is most desirable that these reports should be forwarded to the Local Government Board after submission to the Guardians, so that that Board may have cognisance of those cases where the medical officer's recommendations are persistently ignored by the Guardians.

